# Microscale magnetic field modulation using rapidly patterned soft magnetic microstructures†

**DOI:** 10.1039/d1ra06173a

**Published:** 2021-10-27

**Authors:** Fengshan Shen, Yan Yu, Yuexuan Li, Hongtao Feng, Tianzhun Wu, Yan Chen

**Affiliations:** CAS Key Laboratory of Health Informatics, Shenzhen Institutes of Advanced Technology, Chinese Academy of Sciences Shenzhen China yan.chen@siat.ac.cn

## Abstract

The ability to locally modulate the magnetic field distribution is a prerequisite for efficient manipulation in magnetic force-based microfluidic devices. Here, we report a simple, robust, and fast fabrication method of magnetic microstructures for locally modulating magnetic fields. In the proposed method, a photosensitive magnetic composite consisting of carbonyl-iron microparticles in a poly(ethylene glycol) diacrylate (PEGDA) matrix was utilized to photolithographically fabricate magnetic microstructures. The magnetic behavior of the composite was first evaluated, and then various complicated patterns were fabricated on a glass slide within a few minutes. To demonstrate the capability of magnetic microstructures as a magnetic field concentrator, magnetic microstructures with different orientations to the external magnetic field were designed and fabricated, such as square arrays and grid-like magnetic microstructures. The modulated magnetic fields from such magnetic microstructures were numerically analyzed and then experimentally validated by trapping magnetic hydrogel beads. Further, the magnetically labeled cells were applied to the magnetic microstructures to prove the possibility of cell confinement *via* magnetic guidance in regions that exhibit enhanced magnetic field gradients. Overall, the proposed approach facilitates simple and fast fabrication of soft magnetic microstructures for microscale modulation of magnetic fields, which exhibits an immense application potential in magnetic force-based microfluidic techniques.

## Abstract 2

We present a simple, robust, and ultra-fast method to fabricate soft magnetic patterns for locally modulating magnetic fields.

## Graphical abstract

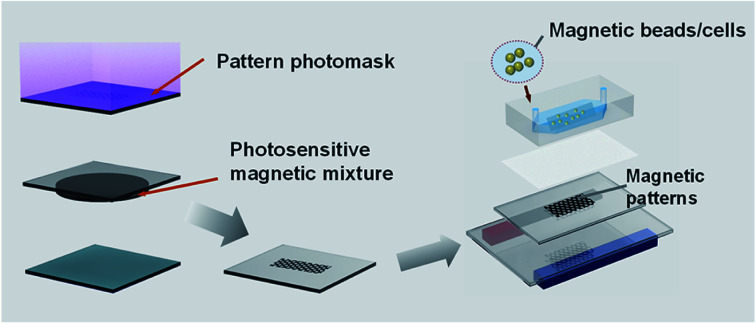

## Introduction

Magnetic force-based microfluidic techniques provide effective platforms for sensitive and precise manipulation of magnetic objects.^1^ In contrast to other alternatives (electrical, optical, and acoustic manipulation), magnetic manipulation offers unique advantages such as simple setup, low cost, insensitivity to temperature or pH, and remote control without direct contact.^2^

Magnetic objects include not only intrinsically magnetic samples such as magnetotactic bacteria,^3^ but also samples that are labelled with magnetic particles.^4^ The use of magnetic particles specifically conjugated to targets offer great selectivity allowing various applications for separation or detection, including immunomagnetic separation of cells,^5^ proteins,^6^ and nucleic acids,^7^ the detection of pathogens.^7^ Besides, with the aid of controllable magnetic forces by designing magnetic fields, cells can guide toward designed spatial arrangements,^8,9^ which make it suitable for the organization of cells such as to align and migrate cells into a targeted pattern^10^ and to pattern cells at specific points.^11^ The generation of flexible and controllable magnetic fields plays a crucial role in making a better use of magnetic guidance.^12^ Generally, the applied magnetic field is generated by permanent magnets positioned external to the microchannel. However, it is challenging to obtain precise control of the magnetic field distribution at the micrometer scale as the dimension of permanent magnets is in the millimeter scale. In addition, a lower magnetic field exerts a weaker magnetic force, and the ability to generate strong magnetic field gradients in the desired area is a prerequisite for efficient and precise magnetic manipulation. It is well established that the strong magnetic field gradients are mainly created at the edges of magnets. Therefore, the high magnetic field area inside the microchannel is mainly limited to the vicinity of the magnet edges. Consequently, the regions farther away from magnet edges appear to be exposed to a low magnetic field. Hence, it is difficult to arbitrarily control the magnetic field distribution in a designated position within the microchannel. To overcome this difficulty, soft magnetic materials in which the flux lines of external fields are concentrated have been introduced into the magnetic force-based microfluidic systems operated by external permanent magnets.^13,14^ Typically, due to their significantly higher magnetic permeability than the surrounding solution, Ni and Ni–Fe alloys are exploited to locally increase the magnetic field gradients.^15,16^ Metal microstructures, such as pillars, strips, and combs with size on the micrometer scale have been fabricated inside or close to the microchannel. Under the applied magnetic fields, these microstructures induce a strong local magnetic field gradient, which is used in several applications, such as the capture and sorting of bacteria or cells and the generation of cell arrays. Although these magnetic microstructures allow the control of magnetic field distribution on the micrometer scale, they are obtained by complex microfabrication techniques like electroplating or sputtering. To generate substantial thickness at the micrometer scale, the processes easily become costly, time-consuming, labour intensive, and generate huge waste.

Alternatively, methods for injecting diluted magnetic particle suspensions, such as nickel microparticle suspensions^17^ or diluted ferrofluid into microchannels have been reported.^18^ However, due to the lower content of magnetic material in such magnetic microstructures, the enhancement effect of the magnetic field gradient is limited. On the other hand, several groups reported the fabrication of magnetic microstructures by molding magnetic composites that were made of PDMS and magnetic particles.^19^ Zhou *et al.* prepared magnetic microstructures by filling and curing a magnetic composite composed of a PDMS matrix doped with carbonyl-iron particles in the microfluidic channel, and they demonstrated the enhanced separation effects of magnetic particles.^17^ Yu *et al.* generated the magnetic structures by filling micro-holes on the mold using nickel powder and then cast it using PDMS to solidify the magnetic pillars that effectively controlled the localized magnetic field distribution.^22^ Although these microstructures showed substantial enhancement of the local magnetic field gradients, the high-density difference between PDMS and magnetic particles easily caused agglomeration and sedimentation of magnetic metal powders during the PDMS curing period.^20,21^ In addition, considering the micrometer-scale mold cavity, where surface tension and viscous drag can dominate, it is not easy to homogeneously fill these microstructures with composites to generate robust magnetic microstructures. The above-mentioned approaches may offer a low-cost alternative to conventional microfabrication approaches; they also require the PDMS-casting cavity fabrication process.

In this study, we propose a rapid photolithographic method to fabricate magnetic microstructures for modulating magnetic fields on a micrometer scale. In this technique, the photosensitive magnetic composite, which is composed of photosensitive poly(ethylene glycol) diacrylate (PEGDA) and carbonyl-iron (CI) microparticle, was deposited by spin coating and rapidly solidified by UV exposure through a photomask. Compared with the micro-molding method using an injection of thermal curable magnetic composites (magnetic particles@PDMS matrix), the use of UV sensitive magnetic composite and the deposition step in the way of spin-coating can dramatically simplify the fabrication process, brining various advantages such as greater accessibility, flexibility, and easy fabrication and ease of integration. Prior to fabrication, the magnetic behaviour of the composite consisting of carbonyl-iron microparticles in the PEGDA matrix was characterized, and the various factors that influence the fabrication pattern fidelity were examined to determine the optimal condition for robust fabrication. To investigate whether it is possible to modulate the magnetic field distribution by varying the shape and orientation of magnetic microstructures, square arrays aligned along the magnetic field direction and grid-like magnetic microstructures arranged obliquely to the magnetic field were designed and fabricated. The modulated magnetic field distribution was numerically analysed and then experimentally validated by trapping magnetic hydrogel beads through magnetic field guidance. Further, the magnetically labelled cells were applied to the magnetic microstructures to prove the possibility of cell confinement in regions exhibiting enhanced magnetic field gradients. This simple and cost-effective approach without requiring the fabrication of casting molding facilitates simple and fast fabrication of soft magnetic microstructures for microscale modulation of magnetic fields, which indicates its immense application potential in magnetic force-based microfluidic techniques.

## Experimental

### Silane functionalization of glass substrates

The glass slides were modified with 3-(trichlorosilyl) propyl methacrylate (TPM; Sigma-Aldrich) using the standard protocol for silane surface modification.^23^ The glass slides were cleaned with a “piranha” solution containing a 3 : 1 mixture of 30% w/v aqueous solutions of H_2_SO_4_ and H_2_O_2_. They were then washed with deionized (DI) water and dried under nitrogen. Subsequently, they were treated in a 1 mM solution of TPM in a 2 : 1 ratio of heptane-carbon tetrachloride for 10 min at room temperature, followed by washing with hexane and water. Both the solution preparation and the silane self-assembly reaction were conducted under a nitrogen atmosphere in a glove bag. The silanized glass slides were stored under a vacuum in a desiccator.

### Preparation of magnetic composite

The magnetic composite was made by CI powder (C3518, Sigma-Aldrich) and PEG-DA (MW 575, Sigma-Aldrich) precursor supplemented with 6% (w/v) photoinitiator 2′-dimethoxy-2-phenylacetophenone (Sigma-Aldrich). The CI powder and PEG-DA precursor solution were thoroughly mixed until a homogeneous material was obtained. To characterize the magnetic properties of CI-PEGDA at various doping ratios, samples with different CI concentrations ranging from 50% to 85.7% w/w were prepared. The magnetization curves of samples with different CI-to-PEG ratios were measured using a vibrating sample magnetometer (VSM; PPMS-9, Quantum Design, USA).

### Fabrication and characterization of magnetic microstructures

**Fig. 1:**
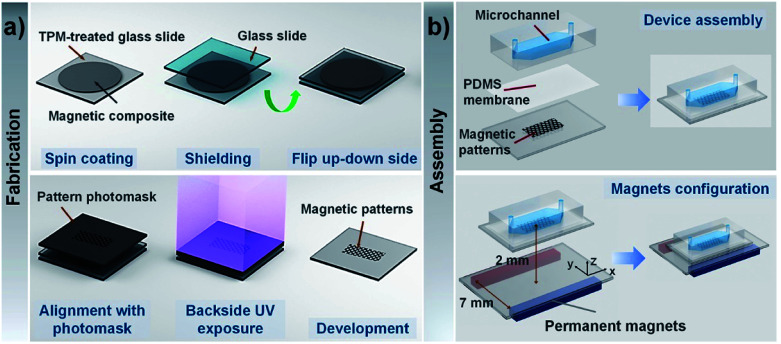
(a) Schematic of the entire fabrication procedure. The mixture of CI and PEG-DA precursor solution was directly spin-coated onto the TPM-treated glass surface. To shield from oxygen, which inhibits photopolymerization by scavenging initiating radicals, the uniform layer of the mixture solution was then covered with another glass slide. Subsequently, the whole set was flipped upside-down, and a photomask with desired features was placed right on the backside of the TPM-treated glass slide. The magnetic composite layer was then exposed to UV light from the backside. Only the regions of CI-PEGDA exposed to UV light underwent free-radical polymerization and were solidified, while during development, unexposed regions of CI-PEGDA were removed from the surface using DI water. (b) Schematic of the chip assembly and configuration of permanent magnets. There is a 2 μm thin PDMS membrane on top of the magnetic microstructure to encapsulate it. Two permanent magnets fixed parallel on a glass slide were exploited to generate an “NS” pole, and they were placed under the microfluidic chip to provide a relatively uniform magnetic field in the vertical direction.

Fig. 1a illustrates the fabrication procedure of CI-PEG microstructures. The mixture of CI and PEG-DA precursor solution was directly spin-coated on the TPM-treated glass surface at 2000 rpm for 15 s. To shield from oxygen, which inhibits photopolymerization by scavenging initiating radicals, the uniform layer of the mixture solution was then covered with another glass slide. Subsequently, the whole set was flipped upside down, and a chrome photomask with desired features was placed right on the backside of the TPM-treated glass slide.

The magnetic composite layer was then exposed to 365 nm UV light from a mask aligner (EVG 610, EV group, Austria) through two layers, namely the photomask and the TPM-treated glass slide. Only the regions of CI-PEGDA exposed to UV light underwent free-radical polymerization and were solidified, while during development, unexposed regions of CI-PEGDA were removed from the surface using DI water and soon dried with nitrogen gas. To examine the influence of the thickness of the TPM-treated glass slide and exposure time on the extent of pattern formation, various thicknesses of the glass slide and exposure time were considered. The morphology of magnetic structures was visualized using a scanning electron microscope (SEM, Zeiss Sigma 300) with an acceleration voltage of 10 kV. The thickness of magnetic structures was measured using a profilometer (DEKTAK 6M, Veeco Instrument, NY, USA).

### Assembly of microfluidic channels

The microfluidic channels were fabricated by the standard soft lithography method. The photoresist SU8 2050 was used to fabricate a master for the channel with a width and depth of 5 mm and 100 μm, respectively. The PDMS mixture (RTV615A : RTV615B = 10 : 1 (w/w)) was poured onto the master mold and baked at 80 °C for 4 h. The PDMS replica was peeled off from the mold and punched for inlets and outlets. Before assembling with a microfluidic chip, to prevent direct contact with samples, a PDMS prepolymer (15 : 1 w/w RTV615A/RTV615B) was spin-coated at 4500 rpm for 45 s to obtain nearly 2 μm-thick thin membrane on top of the magnetic microstructures. Then, the microfluidic channel was bonded to the magnetic microstructures after oxygen plasma treatment (Fig. 1b).

### Numerical simulation of magnetic field

Using COMSOL Multiphysics 5.4, a steady-state model was constructed to simulate the magnetic field distribution in the microfluidic channels integrated with magnetic microstructures. We evaluated three different magnetic microstructure patterns with various sizes and shapes. The patterns of the same size were fabricated with the subsequent magnetic manipulation experiments.

The magnetic flux density (*B*) of permanent magnets and soft magnetic microstructures can be expressed as follows:

For permanent magnets*B* = *μ*_0_*μ*_r_*H* + *B*_r_and for soft magnetic microstructures*B* = *μ*_0_*μ*_r_*H*where *μ*_0_ is the permeability of a vacuum (4 × 10^−7^ H m^−1^), *H* is the magnetic field intensity (A m^−1^), *B*_r_ is the residual flux density of the permanent magnet (1.5 T), and *μ*_r_ is the relative permeability determined for materials. The relative magnetic permeability of air and the permanent magnets were assumed to be 1. The relative magnetic permeability of magnetic microstructures was experimentally determined using the previously reported method. The value was obtained as 2.58, when the doping ratio concentration was 85.7%, and this value was of the same order of magnitude as that for the other magnetic composites.

### Magnetic hydrogel beads

Magnetic hydrogel beads with a diameter of 16 μm, which is in a similar range as that of biological cells, were chosen to test the local increase in the magnetic fields. Owing to the relatively high density of particles, magnetic beads were suspended in Histopaque-119 (Sigma-Aldrich) solution with a concentration of 1.5 × 10^7^ beads per mL, which reduced the particle sedimentation.

### Cell culture and magnetic labelling process

RAW 264.7 cells (murine macrophage-like cell line, CytoBiotech, Guangzhou, China) were cultured in Dulbecco's modified Eagle's medium (DMEM) supplemented with 10% fetal bovine serum (FBS) and 1% penicillin/streptomycin at 37 °C in 5% CO_2_ atmosphere. Before labelling, the magnetic nanoparticles were incubated in a human AB serum medium (Sigma-Aldrich) that generated a protein corona to render nanoparticles more suitable for the intracellular uptake process. For magnetic labelling, 3.6 × 10^4^ cells per cm^2^ were incubated with Ademtech magnetic nanoparticles for 4 h at an iron concentration of 0.75 mM. Then, the cells were stained with SYTO-17 red fluorescent dye for 10 min. Subsequently, the cells were collected, centrifuged, and resuspended at a concentration of 1.5 × 10^7^ cells per mL in Histopaque-1077 (Sigma-Aldrich).

### Magnetic field guidance of magnetic beads/cells

Before introducing magnetic beads/cells, the channel was coated with 10% Pluronic F-68 for 10 min to prevent nonspecific adsorption of particles, and then it was rinsed with Histopaque-1119. Next, 15 μL of magnetic beads/cells were injected into the microfluidic channel using a 1 mL syringe with a syringe pump. Once the magnetic beads were inside the channel, the flow was allowed to stop as no further pressure was applied to the syringe. Two permanent magnets (NdBFe 10 × 10 × 40 mm^3^) fixed parallel on the glass slide were exploited to generate an “NS” pole and were placed under the microfluidic chip to provide a relatively uniform magnetic field. The experimental setup is shown in Fig. 1b, where the gap between the permanent magnets is 7 mm, and the distance from the plane where permanent magnets are fixed to the magnetic microstructures is 2 mm. The magnetic bead patterns were observed using a stereomicroscope (Nikon SMZ18) equipped with a CCD camera (Nikon DS-Ri2). The fluorescence images of the magnetically labelled cells around the magnetic microstructures were acquired by a Leica DFC 360 FX camera mounted on an inverted fluorescence microscope (Olympus IX71).

### Statistical analysis

All the data acquired from experiments were represented as mean ± standard deviation. At least four independent tests were performed to obtain the final results.

## Results and discussion

### Analysis of magnetic composites

**Fig. 2:**
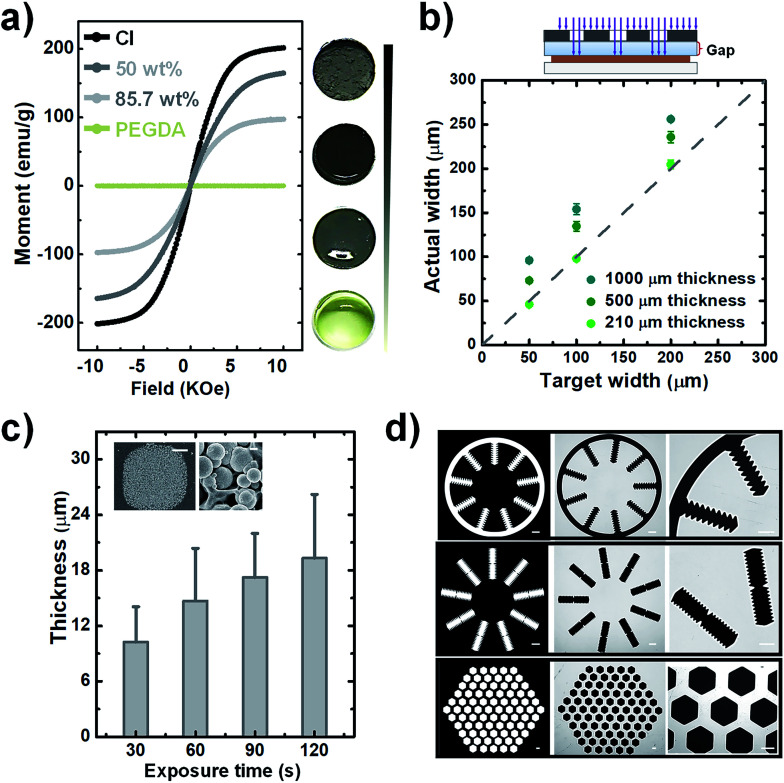
(a) Magnetization curves of CI-PEG composites with different concentrations of CI powder (0%, 50%, 85.7%, and 100%) and the corresponding photographs of samples. (b) The effect of the glass gap on the actual fabricated width. The dotted line shows the region where the actual fabricated width is the same as the target width. (c) Achievable thickness with the variation in the UV dose. The different UV doses were applied with varying exposure times at a constant UV intensity (10 mV cm^−2^). The inset shows the SEM images of the surface of magnetic microstructures. The scale bars are 50 μm (left) and 1 μm (right). (d) Optical patterning of soft magnetic structures with various geometries (left column: photomasks. Middle column: corresponding fabricated magnetic microstructures. Right column: enlarged images of magnetic microstructures). The scale bar is 500 μm.

Prior to the fabrication, the influence of carbonyl-iron microparticle content on the magnetic properties of the composites, samples with carbonyl-iron microparticle content of 0%, 50%, 85.7%, and 100% (w/w) were prepared. The images of these samples with different carbonyl-iron microparticle contents are shown in Fig. 2a. The light-yellow transparent sample is pure PEG (0%) without carbonyl-iron microparticles. As the content of carbonyl-iron microparticles increases, the samples become darker and turn opaque. The uniform color of the composites implies a homogeneous distribution of carbonyl-iron microparticles within the PEG matrix. The magnetization behavior was investigated through VSM measurements, as shown in Fig. 2a. As a diamagnetic material, pure PEG shows a linear magnetization behavior with negligible magnetization. In contrast, composites made by the introduction of carbonyl-iron microparticles into the diamagnetic matrix exhibit significant magnetization. The saturation magnetization decreases with the reduction in the carbonyl-iron microparticles content. In addition, the coercivities and the remnant magnetization are negligible, indicating the soft magnetic property of the composites.^24^ The relative magnetic permittivity of composites was obtained as described in the literature. The value of magnetic permittivity increases from 1.42 to 2.58, when the weight ratio is increased from 50% to 85.7% w/w. The magnetic permittivity values are lower than the value (6.02) for pure carbonyl-iron microparticle powder (100%) but are slightly higher or have the same order of magnitude as those of other magnetic composites such as carbonyl-iron microparticle-PDMS mixtures.^19,20^ Since the higher relative magnetic permittivity leads to an increase in the magnetic susceptibility, which enhances the response to an applied magnetic field, it is better to raise the carbonyl-iron microparticle content even further. However, we found that if the carbonyl-iron microparticle content is increased any further, it is difficult to homogeneously disperse carbonyl-iron microparticles within the matrix due to the drastically increased viscosity. Consequently, we considered the weight ratio of 6 : 1 (85.7%) showing the magnetic permittivity of 2.58 as the optimum ratio to fabricate soft-magnetic structures.

### Fabrication and characterization of soft magnetic microstructure

We verified the feasibility of the proposed photolithographic approach to fabricate magnetic microstructures. For establishing a fabrication procedure, the influence of carbonyl-iron microparticles dispersed within the monomers on the polymerization should be carefully considered. As reported in the literature,^25,26^ magnetic particles inhibit free radical photopolymerization due to their strong UV absorption. In particular, the higher the concentration of magnetic particles in the matrix, the stronger the UV absorption. Therefore, when UV light is exposed from the top of the magnetic composite layer, it is continuously absorbed during the penetration, resulting in a very low UV intensity near the bottom regions. Consequently, a weak crosslinked polymer network is formed close to the glass substrate, so the resulting patterns are not affixed firmly and can be easily peeled off. Therefore, to fabricate patterns in a reproducible and robust way, here, we applied the backside exposure mode. The fabrication process is depicted in detail in Fig. 1a. Initially, the glass slide was functionalized with TPM, which reacts with PEG-DA to covalently anchor magnetic hydrogel onto the glass substrate. The surface anchoring can even prevent delamination from the water-induced swelling of patterns. The backside exposure simultaneously guarantees covalent linkage with TPM and polymerization of monomers close to the glass substrate so that the generated pattern is firmly attached to the glass substrate. In fact, the pattern formed in this way is very robust, so the pattern cannot delaminate even if it is washed with high-pressure streamed DI water during the development.

Even though the backside exposure mode facilitates reproducible operation, the TPM-treated glass slide is sandwiched between the photomask and photosensitive layer, which inevitably induces a glass gap whose length is identical to the glass slide's thickness. Such spacing between the mask and the magnetic photoresist is over orders of magnitude higher than a gap in the lithography proximity mode and thus has a much stronger impact on the pattern's fidelity. Any beams that are not vertically collimated to the photoresist substrate after crossing the photomask can substantially enlarge the exposure zone when they pass through the glass gap. Since the enlargement of the exposure zone may lead to the degradation of pattern fidelity, such as an increase in the width of patterned lines, the effect of the gap needs to be examined. To evaluate the divergence between the target width and the actual fabricated width, pattern lines with widths ranging from 50 to 200 μm were fabricated for testing with varying glass slide thicknesses such as 1000, 500, and 210 μm. The dotted line in Fig. 2b indicates the region where the actual fabricated width is the same as the target width. As expected, a larger gap leads to a greater difference between the target and actual width, while narrowing down the gap with thinner glass slides causes the width of patterns to be close to the target width. Especially in the glass slide whose thickness is 210 μm, the width of patterned lines is approximately equal to the target width, which facilitates sufficient pattern fidelity for the fabrication of magnetic microstructures.

Next, the achievable thickness was evaluated by varying the UV dose. As mentioned earlier, the magnetic composite can greatly reduce the transmittance of UV. The decrease in UV transparency can significantly influence the extent of the curable thickness of the magnetic microstructures. We adjusted the exposure time from 30 to 120 s to increase the UV exposure dose. Fig. 2c shows a notable increase in the cured thickness with the increase in the exposure time from 30 to 60 s, and after that, although the exposure time is increased to 120 s, a slight increase in the achievable height is observed. In addition, a closer inspection of the details of cured magnetic structures by SEM reveals a rough surface because of the embedded particles, and a large number of carbonyl-iron microparticles are affixed in the PEG matrix without much aggregation. The rough surface can be the reason for the large standard deviation of thickness. Although there is a limitation in improving the degree of curing by increasing the UV exposure dose, the achievable thickness obtained here is sufficient to localize the magnetic fields. Thus, in the fabrication process, we selected the exposure time as 120 s and prepared magnetic microstructures with height ranging from 16 to 24 μm for further applications.

Finally, we tested various geometric test patterns with features that included curvature structures, saw-tooth borders, and densely packed honeycomb-like configurations (Fig. 2d). Using these patterns, the corresponding magnetic microstructures were fabricated. It can be seen that even the complicated features of these patterns were successfully transferred to the magnetic composites without defects. The clearly defined magnetic microstructure in the magnified images indicates that the non-crosslinked polymer is completely removed from the surface. Overall, these fabrication results indicate that the magnetic structures can be rapidly fabricated within a few minutes through the proposed simple and robust photolithographic method, no matter how complicated the designed patterns are.

### Controlling the magnetic field distribution in the microchannel

To examine whether the magnetic microstructures can precisely control the magnetic field distribution and then can be used to pattern the magnetic fields for constraining the magnetic objects within the desired areas, we designed two square arrays with different sizes and a grid-like pattern. It is noteworthy that the enhancement of magnetic fields by magnetic microstructures is influenced by the spatial arrangement of patterns with respect to the direction of the external magnetic field. Thus, the two ordered arrays were aligned along the direction of the applied magnetic field, and the grid-like pattern was obliquely placed with respect to the direction of the external field. Firstly, the distribution of magnetic field density gradients of magnetic microstructures was numerically calculated using finite element analysis. These results were experimentally validated by trapping magnetic hydrogel beads. Further, the magnetically labelled cells were also applied to the modulated magnetic field to demonstrate the possibility of cell confinement in desired areas *via* magnetic field guidance. The applied magnetic field is given by the configuration of permanent magnets, as shown in Fig. 1b. Such a configuration offers a nearly uniform magnetic field along the *y*-axis direction (vertical), and the magnetic field in the *x*-axis direction (lateral) is almost zero.^27^ Accordingly, the magnetic microstructures are magnetized by the magnetic field in the vertical direction. Besides, the uniform magnetic field has zero field gradients, and thus the increase in the magnetic field gradient is only attributed to the magnetic microstructures and the enhancement effect can be easily evaluated.

**Fig. 3:**
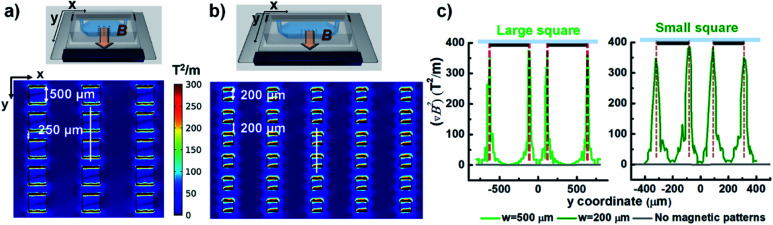
Simulation results of ∇*B*^2^ in the *x*–*y* plane situated 2 μm above the surface of array patterns. (a) Large square pattern array. The 500 × 500 μm^2^ square array has a lateral spacing of 1000 μm and vertical spacing of 250 μm. (b) Small square pattern array. The 200 × 200 μm^2^ square array has a column spacing of 600 μm and row spacing of 200 μm. (c) Characteristic profiles of two adjacent square pattern units along the *y*-axis direction (solid white lines in (a) and (b)). For comparison, the simulation results in the absence of patterns are displayed as grey lines.

For square patterns, the spatial arrangement is as follows: the patterns with sizes of 500 × 500 μm^2^ and 200 × 200 μm^2^ are vertically arrayed along the direction of the external magnetic field, and the vertical spacing between patterns was designed to be 250 μm and 200 μm, which corresponded to a half and the total size of the pattern, respectively. The horizontal spacing between the adjacent lanes of the respective array was set to 1000 μm and 600 μm, which is as large as three-fold and two-fold of each pattern size. When magnetized by the external magnetic field, the magnetic microstructures generate a strong localized field to provide an enhanced magnetic field density gradient (∇*B*^2^), which is proportional to the magnetic force acting on the magnetic particles. Fig. 3a and b show the distribution of magnetic field density gradient in these spatial arrangements of patterned arrays. Obviously, ∇*B*^2^ is dramatically increased in just the areas with patterns in the vertical direction (but not in the horizontal direction) that are perpendicular to the magnetic field. Additionally, in the regions without patterns, there is no apparent increase and ∇*B*^2^ becomes negligible. Meanwhile, the characteristic profile of ∇*B*^2^ generated by the two adjacent pattern units along the *y*-axis is displayed together with that without the magnetic microstructures for a comparison (see Fig. 3c). The value of ∇*B*^2^ is negligible in the absence of magnetic microstructures. However, applying square magnetic microstructures causes a significant change in the magnetic field distribution at the pattern boundary regions, leading to a sharp increase in the gradient in these regions. Nevertheless, this effect rapidly diminishes with distance. Consequently, the value of ∇*B*^2^ is maximum at the edge and shows four peaks at the edge. The magnitude of enhancement is as high as 400 T^2^ m^−1^ as compared to 1.2 T^2^ m^−1^ in the absence of magnetic microstructures, which are able to provide up to 400 times stronger magnetic force. However, it decays rapidly and then becomes insignificant at a distance of 125 μm from the edge, making it difficult to realize a reinforcement effect by magnetic microstructures beyond that distance. Conversely, when the width between the edges is closer than their influential limit distance, the local gradients created by edges can overlap, enlarging the value of ∇*B*^2^ along the width between edges. This explains why the profile with the pattern size of 200 μm with a 200 μm spacing width can always exhibit a higher value of ∇*B*^2^ than that without patterns. In contrast, for the pattern size of 1000 μm with an adjacent width of 250 μm, the value remains slightly higher in the adjoining width range. However, near the center of the pattern, it can drop to a low level, almost equal to the level without magnetic microstructures.

**Fig. 4:**
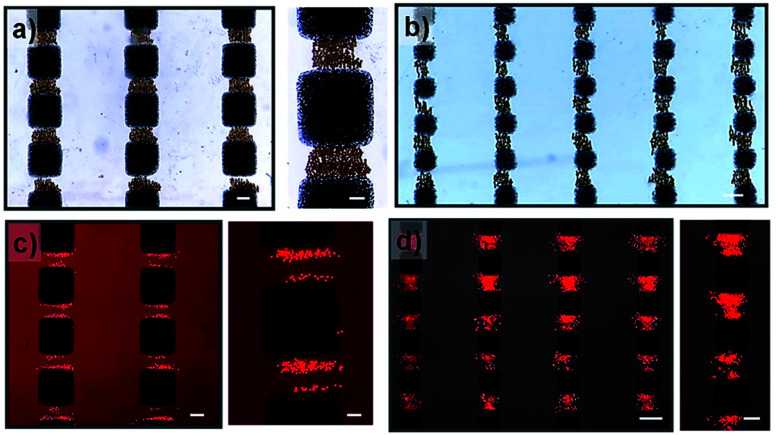
Hydrogel magnetic beads captured in, (a) large square pattern array and (b) small square pattern array. The magnetically labelled cells are constrained d in the (c) large square pattern array and (d) small square pattern array. The scale bar is 200 μm. The scale bars in enlarged images are 100 μm.

To experimentally validate the simulation results, the magnetic hydrogel beads were trapped under modulated magnetic field. The magnetic hydrogel beads in suspension are introduced into the microchannel integrated with magnetic microstructures. After filling, the flow is arrested for excluding other influences, such as hydrodynamic force, other than the magnetic force. There are generally two types of magnetic forces experienced by magnetic beads in external magnetic fields, originating from their permanent or induced dipole moments: the interaction forces between magnetic particles and magnetic fields and the interparticle interaction between magnetic particles.^28^ When an external magnetic field is applied, magnetic hydrogel beads, which were initially randomly distributed in the channel, are attracted to a nearby high magnetic field area due to the magnetic force induced by the external magnetic fields. Consequently, magnetic beads are locally trapped in the magnetic microstructure regions, in which the magnetic field gradient is enhanced, thereby generating patterns of magnetic hydrogel beads according to the magnetic microstructures. Fig. 4a and b show magnetic bead patterns generated by the square pattern arrays. It was observed that the magnetic hydrogel beads are trapped only in the adjacent areas between two patterns along the *y*-axis direction, where the periodic edge regions appear, which have an increased field gradient and accordingly exert a strong magnetic force. Simultaneously, there are no magnetic beads in the region devoid of magnetic microstructures as well as in the adjoining areas along the *x*-axis direction. The magnetic force in these regions is negligible due to the almost zero field gradient from a relatively uniform magnetic field. In addition, the captured magnetic beads are not clustered. Instead, due to the magnetic dipole interactions between them, they form chain-like agglomerates oriented in the field direction. We further attempted the magnetic guidance of cells labelled with magnetic nanoparticles under modulated magnetic field. As shown in Fig. 4c and d, the magnetically labelled cells are locally confined to the high magnetic field zones of square patterns. For the small square pattern array, the cells are distributed both around the edges or in the area between the patterns. On the other hand, in the large square pattern array, the cells are only closely arranged near the edges. These results are in excellent agreement with the simulation results, which indicates that the smaller pattern shows a higher field gradient value between the edges due to the close overlap. However, unlike the trapped magnetic beads assembled to chain-like agglomerates, the captured cells do not show any effective response of magnetic dipole interaction between them due to their negligible induced magnetic moments, indicating that the amount of magnetic loading in the cells is much lower than that in the magnetic beads. Moreover, the cells are exposed to a much smaller magnetic force than the magnetic beads, even under the same strength of magnetic field gradients.

**Fig. 5:**
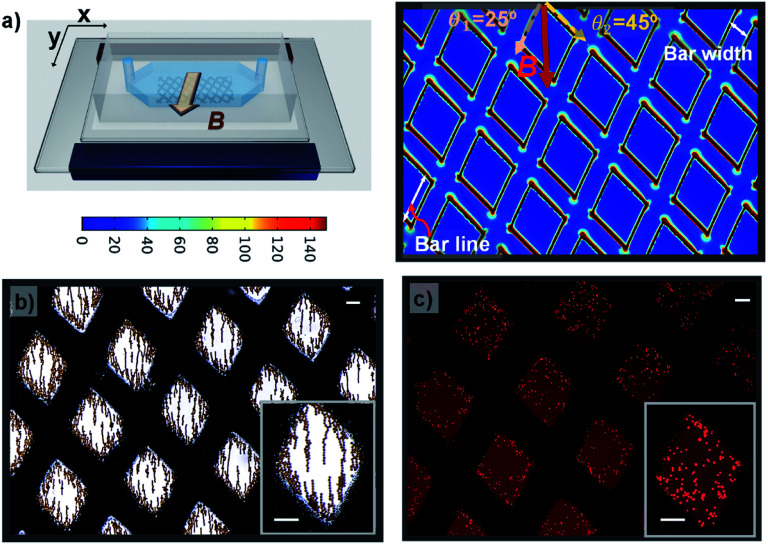
(a) Simulation results of (∇*B*^2^) in the *x*–*y* plane situated 2 μm above the surface of the grid-like pattern. (b) Hydrogel magnetic beads captured in the grid-like pattern. (c) Magnetically labelled cells are constrained within the grid-like pattern. The scale bars represent 200 μm.

Next, a grid-like pattern with diamond-shaped holes of size 650 μm and grid bar width of 320 μm was fabricated. The grid-like pattern was obliquely arranged with respect to the magnetic field direction, creating angles of 25° and 45° between the magnetic field and the right and left lines of the grid, respectively. The simulation result of ∇*B*^2^ in the plane situated 2 μm above the surface of the grid-like pattern is shown in Fig. 4a. The areas without the grid-like pattern exhibit negligibly low values of ∇*B*^2^. In the patterned area, the inner area of the grid shows no increase in intensity, but on the border, the value is dramatically increased. As expected, it is maximal at the edges and sharply decays away from the edges. Interestingly, the border regions of the grid bars with a larger angle to the external magnetic field show higher values of ∇*B*^2^ than the border zones of grid bars with a smaller angle. These discrepancies between the bars with different angles are likely due to the difference in the distribution of the magnetic field in these bars. When the grid-like pattern acts as a magnetic concentrator, the external magnetic field is preferentially incident on to the bars with a smaller angle of incidence, and the magnetic field lines are completely confined in the areas, leading to a uniform magnetic field density distribution in such bar areas. By contrast, the bars with a larger angle of incidence show spatially non-uniform field distribution due to the less concentrated field lines in the bar regions (see Fig. S1†). For the trapping of magnetic hydrogel beads, as shown in Fig. 2b, it is observed that the magnetic hydrogel beads are confined only to the hole areas in two different distributions depending on their location. Briefly, near the boundary regions, most of the beads attached to the borderlines have the maximum magnitude of the magnetic field gradient. Further, there are no beads above the magnetic microstructures bar areas. This is because the beads initially distributed in the bar areas migrate toward the borderlines and later tend to be adhered there due to the strong magnetic force. The beads that are relatively far from the boundary are not affected by the magnetic force produced by the boundary regions. In fact, these beads are themselves magnetized by the external magnetic field and form a chain arranged in the direction of the magnetic field through the magnetic dipole interaction between them. When this chain is long enough to extend to the boundary, it can be seen that the entire chain migrates to the borderlines and adheres to there. These observations are consistent with the simulation results, which validate the modulation of the local magnetic field by the magnetic microstructure. On the other hand, the cells located in the hole areas are not affected by the external magnetic field. Only the cells initially situated within the bar area receive a sufficiently strong magnetic force and appear to move toward the nearby borderlines. Consequently, the confinement of cells is observed only as islands in the hole areas (see Fig. 5c). Therefore, it can be concluded that it is possible to distribute the cells in the desired areas through magnetic guidance under a controllable magnetic field distribution created by the designed magnetic microstructures.

## Conclusions

In this study, a photolithographic technique was proposed for the fabrication of magnetic microstructures that generated tunable locally enhanced magnetic field gradients using a photosensitive polymer, which was loaded with a high content of carbonyl-iron microparticles. We characterized the magnetic behavior of the composite consisting of carbonyl-iron microparticles in the PEGDA matrix and demonstrated that the magnetic composite has the ability to control local magnetic fields. To investigate the precise control of the magnetic field distribution, we designed and fabricated square arrays with different sizes as well as a grid-like pattern. The distribution of the magnetic field density gradients of these magnetic microstructures was numerically calculated using finite element analysis. Further, the simulation results were experimentally validated by trapping magnetic hydrogel beads under modulated magnetic fields. In addition, the magnetically labelled cells were applied to the modulated magnetic field to demonstrate the possibility of cell confinement in the desired areas with magnetic field guidance. Furthermore, the combination of recent progressive development to considerably increase the permeability by using anisotropic agglomerates of magnetic particles in the polymer matrix can hold promises for further increase in performances of patterned magnetic microstructures.^29,30^ Overall, the proposed approach allows simple, robust, and rapid fabrication of any complicated shape of magnetic microstructures for the microscale modulation of magnetic fields, showing an immense application potential in magnetic force-based microfluidic techniques.

## Author contributions

Fengshan Shen: conceptualization, designing devices, methodology, investigation, performing experiments, data analysis, writing original draft and editing. Yu Yan and Yuexuan Li: performing experiments. Hongtao Feng and Tianzhun Wu: analysing, investigation, data analysis. Yan Chen: conceptualization, supervision, methodology, writing review and editing.

## Conflicts of interest

There are no conflicts to declare.

## Supplementary Material

RA-011-D1RA06173A-s001
