# Supplementary material for: Microscale magnetic field modulation using rapidly patterned soft magnetic microstructures
Source: RSC Adv. 2021 Oct 27;11(55):34660–8. doi: 10.1039/d1ra06173a (PMC9042693; doi:10.1039/d1ra06173a)
Supplement: RA-011-D1RA06173A-s001 [file RA-011-D1RA06173A-s001.pdf]

Electronic Supplementary Material (ESI) for RSC Advances.
This journal is © The Royal Society of Chemistry 2021

Electronic Supplementary Information for

Microscale magnetic field modulation using ultra-rapidly fabricated soft magnetic patterns

*Fengshan Shen, Yan Yu, Yuexuan Li, Hongtao Feng, Tianzhun Wu and Yan Chen**

CAS Key Laboratory of Health Informatics, Shenzhen Institutes of Advanced Technology, Chinese Academy of Sciences, Shenzhen, China

***Corresponding author: Yan Chen**

E-mail: yan.chen@siat.ac.cn

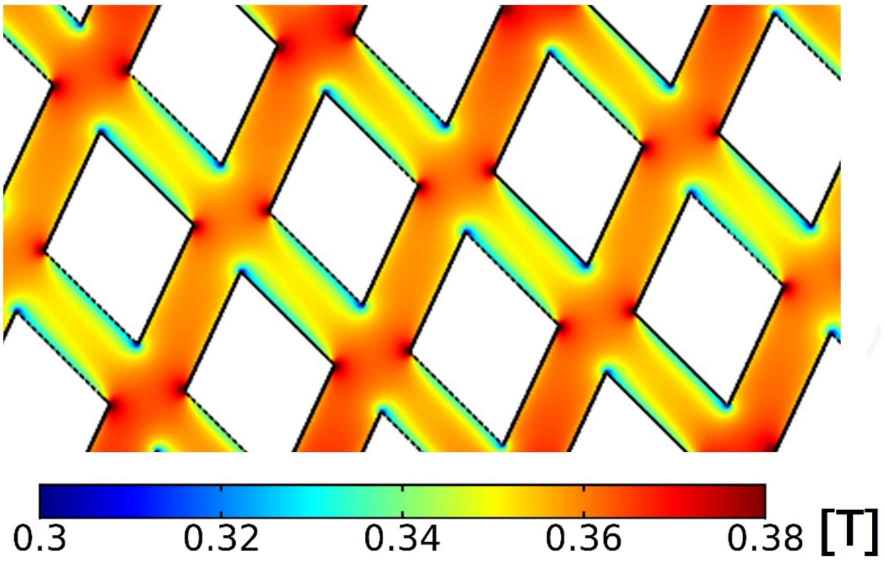

Fig. S1. Numerical simulation of the magnetic field distribution in the grid-like pattern. There is discrepancy in the distribution of the magnetic field in bar areas with different angles.
